# Stroke volume changes induced by a recruitment maneuver predict fluid responsiveness in patients with protective ventilation in the operating theater

**DOI:** 10.1097/MD.0000000000004259

**Published:** 2016-07-18

**Authors:** Bruno De Broca, Jeremie Garnier, Marc-Olivier Fischer, Thomas Archange, Julien Marc, Osama Abou-Arab, Hervé Dupont, Emmanuel Lorne, Pierre-grégoire Guinot

**Affiliations:** a Anesthesiology and Critical Care Department, Amiens University Medical Center, Place Victor Pauchet, Amiens; b Pôle Réanimations Anesthésie SAMU/SMUR, CHU de Caen, Caen; c INSERM U1088, Jules Verne University of Picardy, Amiens, France.

**Keywords:** anesthesia, preload indicator, protective mechanical ventilation, volume expansion

## Abstract

During abdominal surgery, the use of protective ventilation with a low tidal volume, positive expiratory pressure (PEEP) and recruitment maneuvers (RMs) may limit the applicability of dynamic preload indices. The objective of the present study was to establish whether or not the variation in stroke volume (SV) during an RM could predict fluid responsiveness.

We prospectively included patients receiving protective ventilation (tidal volume: 6 mL kg^−1^, PEEP: 5–7 cmH_2_O; RMs). Hemodynamic variables, such as heart rate, arterial pressure, SV, cardiac output (CO), respiratory variation in SV (ΔrespSV) and pulse pressure (ΔrespPP), and the variation in SV (ΔrecSV) as well as pulse pressure (ΔrecPP) during an RM were measured at baseline, at the end of the RM, and after fluid expansion. Responders were defined as patients with an SV increase of at least 15% after infusion of 500 mL of crystalloid solution.

Thirty-seven (62%) of the 60 included patients were responders. Responders and nonresponders differed significantly in terms of the median ΔrecSV (26% [19–37] vs 10% [4–12], respectively; *P* < 0.0001). A ΔrecSV value more than 16% predicted fluid responsiveness with an area under the receiver-operating characteristic curve (AU) of 0.95 (95% confidence interval [CI]: 0.91–0.99; *P* < 0.0001) and a narrow gray zone between 15% and 17%. The area under the curve values for ΔrecPP and ΔrespSV were, respectively, 0.81 (95%CI: 0.7–0.91; *P* = 0.0001) and 0.80 (95%CI: 0.70–0.94; *P* < 0.0001). ΔrespPP did not predict fluid responsiveness.

During abdominal surgery with protective ventilation, a ΔrecSV value more than 16% accurately predicted fluid responsiveness and had a narrow gray zone (between 15% and 17%). ΔrecPP and ΔrespSV (but not ΔrespPP) were also predictive.

## 1 Introduction

Early goal-directed fluid therapy during surgery decreases the length of hospital stay and postoperative morbidity, and its use at the bedside is recommended.^[1,2]^ This approach is based on fluid optimization and the maximization of cardiac output (CO) by using dynamic preload indices or fluid titration. Dynamic preload indices (such as the respiratory variation in pulse pressure (ΔrespPP) or stroke volume [ΔrespSV]) are based on the interaction between the cardiovascular and pulmonary systems during positive-pressure mechanical ventilation.^[3]^ Nevertheless, a low tidal volume (TV), heart rhythm disorders, and right heart failure decrease the reliability of these indices.^[4]^ Protective ventilation (with a low TV, positive expiratory pressure (PEEP), and the use of a recruitment maneuver [RM]) appears to be associated with a shorter stay in hospital and a lower complication rate.^[5–7]^ However, protective ventilation alters the predictability of dynamic preload indices and thus limits their use in the operating theatre.^[8]^ Other preload tests (such passive leg-raising and an end-expiratory maneuver) can be used but are less accurate and more difficult to implement.^[9]^ In this context, variations in the CO or stroke volume (SV) during the RM could also be monitored as a guide to preload dependency. RMs increases intrathoracic pressure, which in turn causes a transient decrease in CO and arterial pressure; this may depend on preload status.^[10]^ This approach has been studied and validated in septic patients, but not in patients with protective ventilation.^[11]^ The main objective of the present study was to establish whether or not the variation in SV during a RM (ΔrecSV) predicts a further increase in SV upon fluid expansion. We also assessed the predictive value of ΔrespPP, ΔrespSV and the variation in pulse pressure during a RM (ΔrecPP) in this context.

## 2 Methods

### 2.1 Ethics

After approval by the local investigational review board (*Comité de Protection des Personnes Nord-Ouest II*, Amiens, France; reference: 2014–79), we performed a prospective study in the operating theater at Amiens University Medical Center between January and September 2015. All patients received written information on the study's objectives and procedures, and gave their informed consent to participation before surgery. The present manuscript was drafted in compliance with the STROBE checklist for cohort studies.^[12]^

### 2.2 Patients

The main inclusion criteria were as follows: invasive arterial blood pressure and esophageal Doppler monitoring (EDM), the use of protective ventilation, and fluid challenge in the operating theater. Patients with frequent ectopic beats, preoperative arrhythmia, right ventricular dysfunction, or spontaneous ventilation were not included. Fluid expansion was performed in patients with arterial hypotension (ie, a systolic arterial pressure below 100 mm Hg) or an SV decrease of more than 10%. In our institution, fluid challenge consists of infusion of 500 mL of Ringer lactate via a pressure bag. Patients having undergone several RMs were included only once (ie, when they first met the inclusion criteria).

### 2.3 Anesthesia

Each patient was monitored with pulse oximetry, arterial invasive blood pressure monitoring, and 5-lead electrocardiography. Balanced general anesthesia was applied. All patients were intubated and ventilated in volume-controlled mode. The exact choice of drug was left to the anesthetist's discretion (either propofol or etomidate as hypnotics and either remifentanil or sufentanil as opioids). Anesthesia was maintained with either an inhaled hypnotic (desflurane or sevofurane) or propofol and the opioid used for induction. Neuromuscular blockade was systematically induced with rocuronium (0.6 mg kg^−1^) or cisatracurium (0.15 mg kg^−1^). The TV was adjusted to the ideal body weight to obtain 6 mL kg^−1^, the ventilatory rate was set in order to maintain an end-tidal CO_2_ pressure of 35–37 cmH_2_O, and PEEP (5–7 cmH_2_O) was applied. Static pulmonary compliance was calculated as TV divided by (pressure plateau minus total end expiratory pressure). Epidural anesthesia was used after surgery, but was never used during surgery.

### 2.4 Esophageal Doppler monitoring

The EDM probe (CardioQ, Deltex Medical, Laboratoire Gamida, Eaubonne, France) was positioned so as to obtain the optimum signal for blood velocity in the descending aorta. SV and CO were calculated continuously (beat-by-beat) from the aortic blood flow velocity by using EDM software, and the values were averaged over a 10-second moving window. Respiratory variations (Δresp) of EDM values were obtained as described previously, regardless of the respiratory cycle.^[13]^ All measurements were analyzed off-line using a video of the monitoring signal. We calculated the mean ± standard deviation (SD) intraobserver and interobserver reproducibility (2.8 ± 1.3% and 5.1 ± 3.2%, respectively).

### 2.5 The recruitment maneuver

RMs were performed as described in the literature^[5–7]^; immediately after intubation, every 30 minutes during the procedure, before extubation, and whenever the ventilatory circuit was disconnected. In the absence of a literature consensus on the type of intraoperative RM to be used in patients with normal (healthy) lung compliance, the RM consisted of the application of a continuous positive inspiratory pressure of 25 cmH_2_O for 25 seconds (as implemented in our center's department of anesthesia).

### 2.6 Data collection

We recorded demographic data (weight, age, gender, ASA (American Society of Anesthesiology) score, medical history, and the type of operation), respiratory parameters (TV, respiratory rate, insufflation pressure [peak], plateau pressure, and the level of PEEP), and hemodynamic parameters (systolic blood pressure [SAP], diastolic blood pressure [DAP], mean arterial pressure [MAP], heart rate, ΔrespPP, ΔrespSV, and EDM data [SV and CO]).

### 2.7 Study protocol

When a patient met the inclusion criteria, the investigating physicians collected a first set of demographic, ventilatory, and hemodynamic data (Base 1) before fluid expansion. After the RM had been performed, a second dataset (RM) was recorded. A third dataset was recorded 5 minutes after all the hemodynamic variables had returned to baseline values (Base 2). Next, fluid expansion (500 mL of Ringer lactate over 10 minutes via a pressure bag) was performed. On the basis of very sparse literature data on the maximum possible effect of fluid expansion on hemodynamic variables, we decided to acquire a fourth and last set of hemodynamic data (FE) 5 minutes after the fluid expansion.^[9,11,13]^

ΔrespPP was automatically calculated by the Philips monitoring system; this method has already been validated.^[14]^ ΔrespSV was calculated as described previously: ΔrespSV = ([SVmax − SVmin]/[SVmax + SVmin])/2 × 100, where SVmin and SVmax are the minimum and maximum SV values over a single respiratory cycle, respectively.^[13]^ All values correspond to the mean of 3 measurements. ΔrecPP was calculated as follows: ΔrecPP = (PP_base1_ – PP_RM_)/(PP_base1_) × 100. ΔrecSV was calculated as follows: ΔrecSV = (SV_base1_ – SV_RM_)/(SV_base1_) × 100, where SV_RM_ is the mean of the last 3 SVs at the end of the RM. ΔrecSV and ΔrecPP are expressed as absolute values.

Nonresponders and responders were defined with regard to the change in SV (expressed as a percentage) after fluid expansion.^[15]^ A positive response (fluid responder) was defined as an SV increase of at least 15% between Base 2 and fluid expansion.^[15]^ This cut-off was chosen in accordance with the literature data on fluid expansion and because it is twice the value of the interobserver/intraobserver reproducibility of SV measurements using EDM.

### 2.8 Statistics

Based on a pilot study of 15 patients, it was calculated that a sample size of 60 patients would be sufficient to demonstrate that (i) ΔrecSV can predict fluid responsiveness with an area under the curve (AUC) of between 0.8 and 0.87, a power of 80%, an alpha risk of 0.05 and a beta risk of0.2, and (ii) ΔrecSV is correlated with SV changes upon fluid expansion (with a ratio ranging from 0.5 to 0.7). The data distribution was assessed using a D’Agostino–Pearson test. Data are expressed as the number (percentage), the mean (SD) or the median (25–75^le^) as appropriate. The nonparametric Wilcoxon rank sum test, Student's paired *t* test, Student's *t* test, the Mann–Whitney test, the Kruskal–Wallis test and an analysis of variance with Bonferroni *post hoc* correction were used to assess statistical significance, as appropriate. Categorical variables were compared in a chi-square test or Fisher exact test. Linear correlations were tested using Pearson rank method. A receiver-operating characteristic (ROC) curve was drawn for ΔrecSV, ΔrespSV, ΔrespPP, and ΔrecPP. We selected the threshold that gave the highest Youden index. The gray zone (corresponding to 2 cut-offs between which the prediction of fluid responsiveness remained uncertain) was calculated using 3 response classes (negative, inconclusive, and positive). Inconclusive responses were cut-off values with a sensitivity and a specificity below 90% (ie, diagnostic tolerance of 10%).^[17]^ The method described by DeLong et al was used to compare the areas under the ROC curve (AUC) associated with the variables.^[16]^ The association between the volume of fluid infused (mL kg^−1^), cardiovascular variables (heart rate, SAP, MAP, DAP, SV, CO, ΔrespSV, ΔrecSV, and ΔrecPP) and fluid responsiveness was assessed using a univariate logistic regression model. Variables with a *P* value less than 0.10 in the univariate model were included in a multivariate logistic regression model with backward selection. The threshold for statistical significance was set to *P* < 0.05. Statistical analyses were performed with SPSS software (version 22, IBM, New-York, USA) and R software (version 3.3.1, Vienna, Austria) with the ROCR package.

## 3 Result

**Figure 1:**
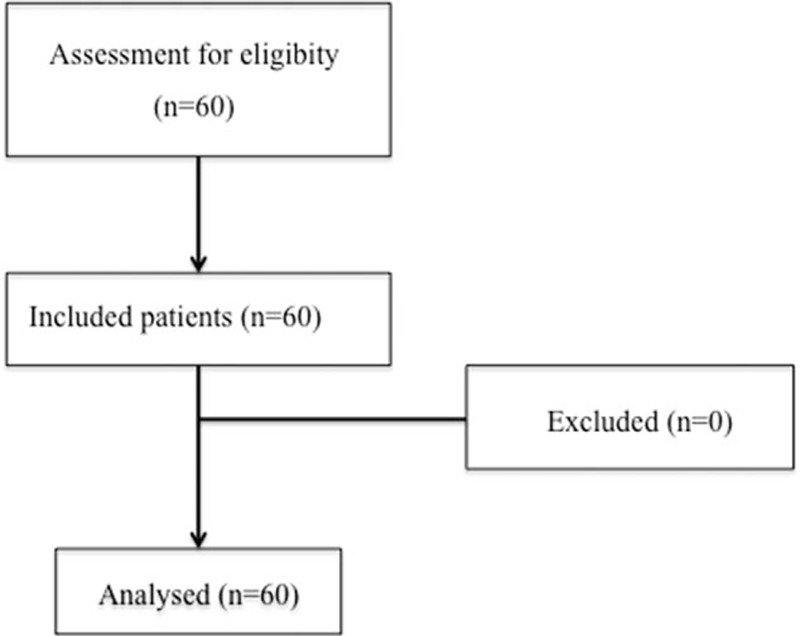
Study flow chart.

A total of 60 patients were included. The study flow chart is shown in Fig. 1. Baseline characteristics for the whole study population are reported in Table 1. During the study period, none of the patients had received continuous infusions of norepinephrine or dobutamine or boluses of ephedrine.

**Table 1:**
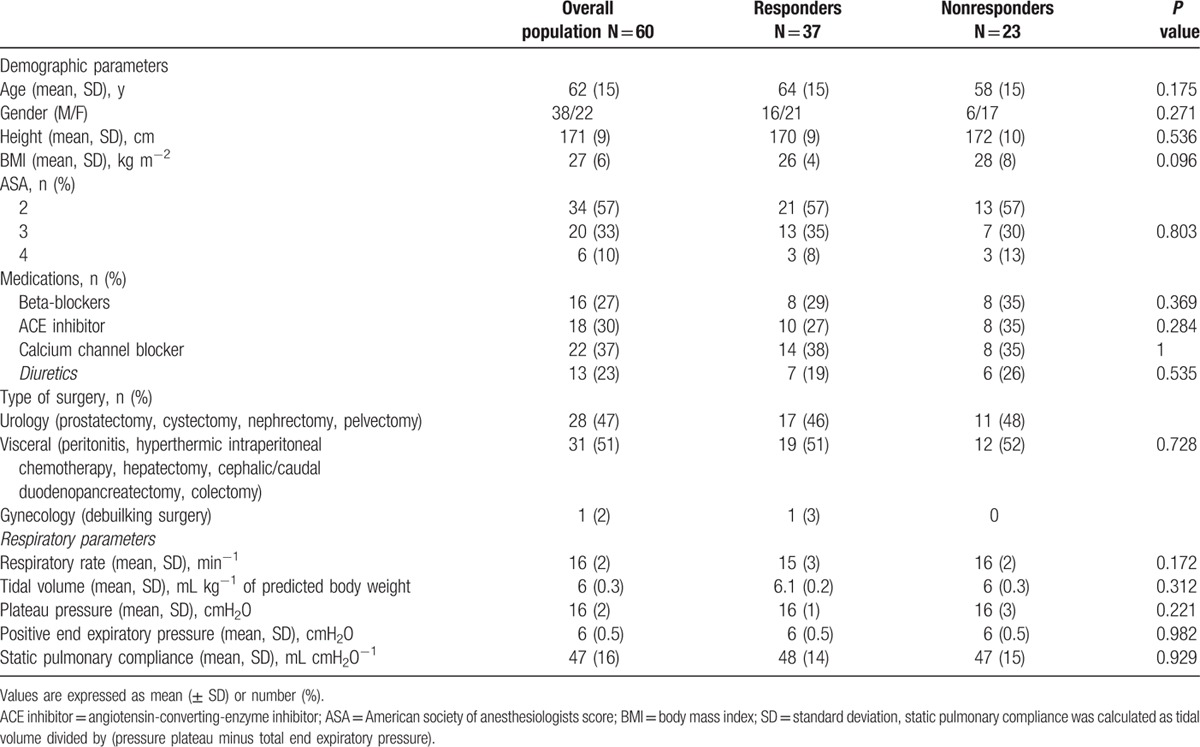
Demographic characteristics of the study population.

Thirty-seven (62%) of the 60 patients were classified as fluid responders, and thus the remaining 23 (38%) were classified as non-responders. The mean ± SD volume used in the fluid challenge was similar in the responders and nonresponders (6.9 ± 1.7 mL kg^−1^ vs 6.5 ± 1.6 mL kg^−1^, respectively; *P* = 0.225). The baseline SV and CO were lower and ΔrespSV was higher in responders than in nonresponders (Table 2). Fluid expansion increased MAP, SV, and CO and decreased ΔrespSV and ΔrespPP in responders only (Table 2). Responders and nonresponders differed in terms of the median SV increase of SV with fluid expansion (23% [18–33] vs 6 % [3–11], *P* < 0.05). The median ΔrecSV was greater in fluid responders than in nonresponders patients (26% [19–37] versus 10% [4–12], respectively; *P* < 0.05); the same was true for ΔrecPP (29% [16–38] vs 9% [3–20], respectively; *P* < 0.05; Table 2).

**Table 2:**
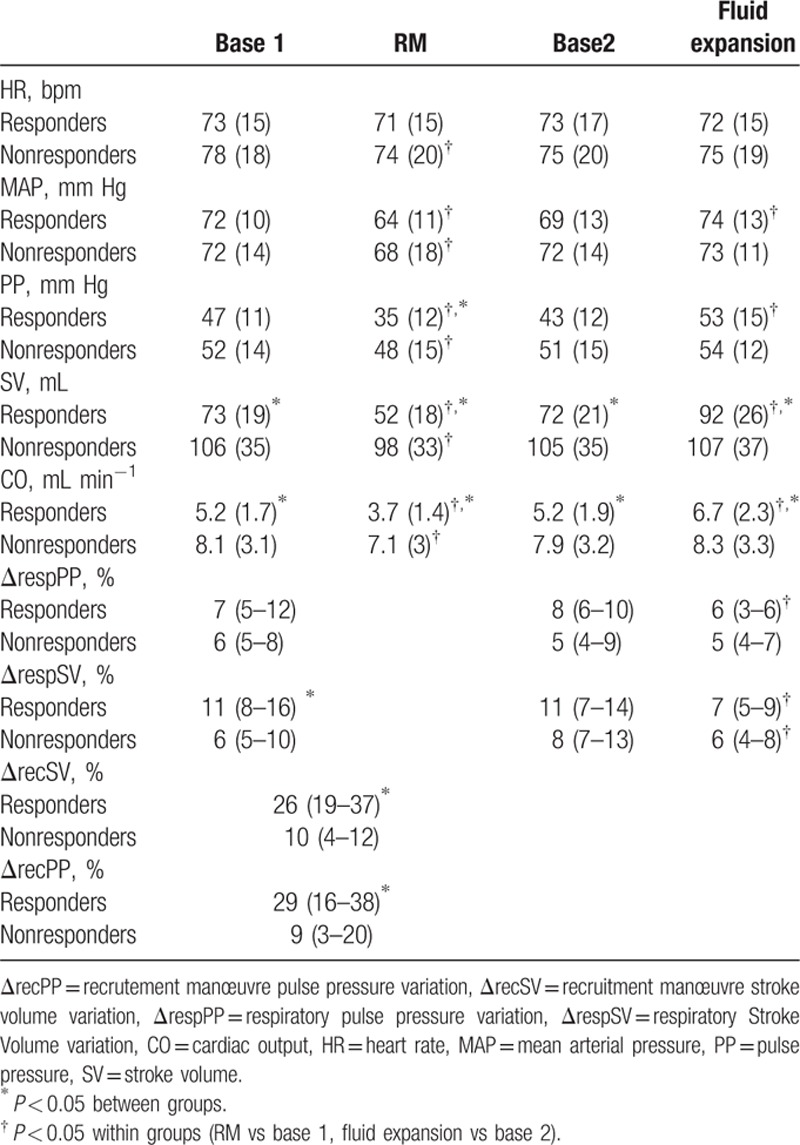
Cardiovascular variables in responders and nonresponders expressed as mean (SD) or median (25–75^le^).

ΔrecSV and ΔrecPP were correlated with SV variations upon fluid expansion (*r* = 0.63 [95%CI: 0.44–0.76], *P* < 0.0001, and *r* = 0.48 [95%CI: 0.26–0.66], *P* < 0.0001, respectively). Baseline ΔrespSV and ΔrespPP were also correlated with SV variations upon fluid expansion (*r* = 0.541 [95%CI: 0.29–0.68], *P* = 0.0001, and *r* = 0.42 [95%CI: 0.16–0.62], *P* = 0.002, respectively).

**Figure 2:**
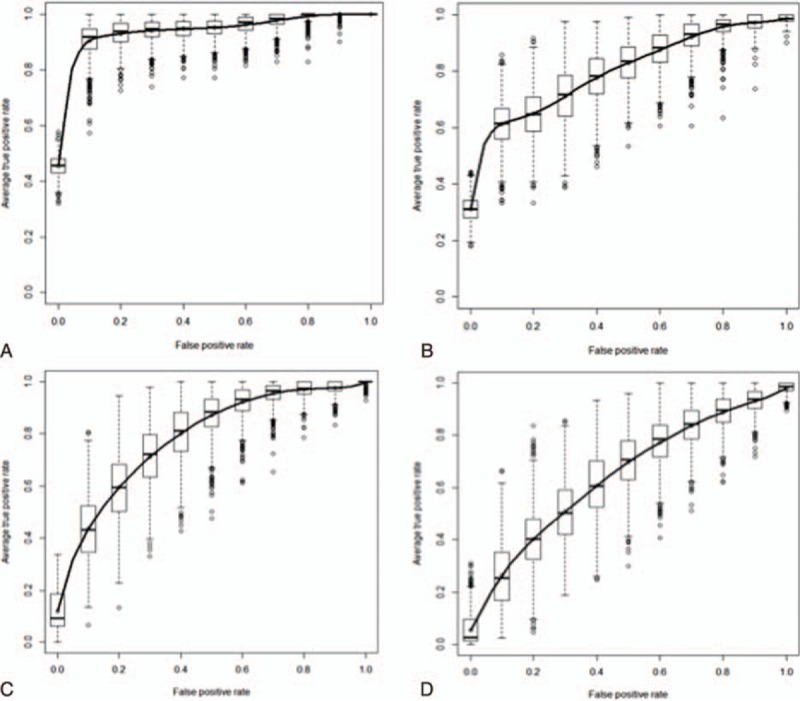
Receiver operating characteristic curves for the SV variation during an recruitment maneuvers (ΔrecSV: 0.95 [95%CI: 0.91 to 0.99], *P* < 0.0001) (A), the pulse pressure variation during an recruitment maneuvers (ΔrecPP: 0.81 [95%CI: 0.7–0.91], *P* < 0.0001) (B), the respiratory variation in stroke volume (ΔrespSV: 0.80 [95%CI: 0.7–0.94], *P* = 0.01) (C), and the respiratory variation in pulse pressure (ΔrespPP: 0.65 [95%CI:0.51–0.77], *P* = 0.07) (D), with a view to discriminating between fluid expansion responders and nonresponders.

ΔrecSV was strongly predictive of fluid responsiveness, with an AUC of 0.95 (95%CI: 0.91–0.99, *P* < 0.0001). The gray zone ranged from 15% to 17%. ΔrecPP predicted fluid responsiveness with an AUC of 0.81 (95%CI: 0.7–0.91, *P* = 0.0001). The gray zone ranged from 6% to 26%. ΔrespSV predicted fluid responsiveness with an AUC of 0.80 (95%CI: 0.70–0.94, *P* = 0.0001). The gray zone ranged between 6% and 11%. ΔrespPP was not predictive of fluid responsiveness, and yielded an AUC of 0.65 (95%CI: 0.51–0.77) (*P* = 0.07, Table 3, Fig. 2). The AUC of ΔrecSV was greater than those of ΔrecPP and ΔrespSV (*P* < 0.05). The AUCs of ΔrecPP and ΔrespSV were not different.

**Table 3:**
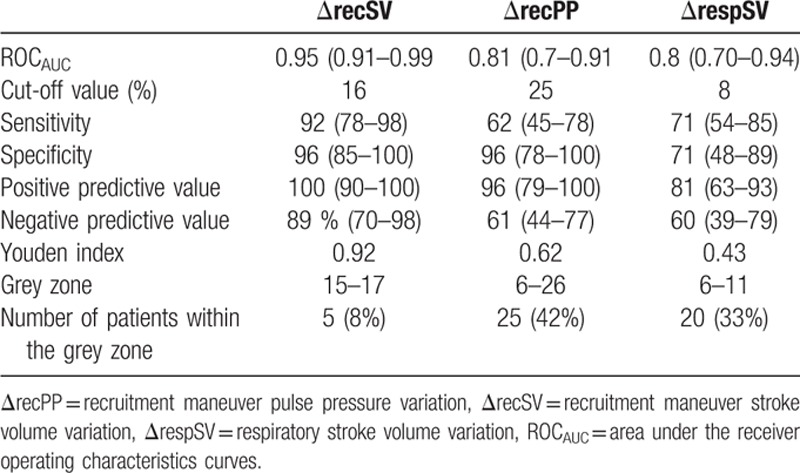
Diagnostic performance of ΔrecSV, ΔrecPP, and ΔrespSV to predict fluid responsiveness.

In a multivariate logistic regression analysis, ΔrecSV was found to be the only factor significantly associated with fluid responsiveness (odds ratio = 1.5 [95%CI: 1.13–2.1], *P* < 0.0001).

## 4 Discussion

Our present results demonstrate that the SV changes induced by an RM (ΔrecSV) predict fluid responsiveness in patients receiving protective ventilation during surgery. A ΔrecSV more than 16% during RM predicted fluid responsiveness and had a narrow gray zone (between 15% and 17%). The ΔrecPP and ΔrespSV were also predictive of fluid responsiveness in patients with protective ventilation. In contrast, ΔrespPP was not predictive of fluid responsiveness in this setting.

To understand the impact of the RM on the cardiovascular system, several physiological aspects must be taken into account. Firstly, none of our patients had pulmonary disease. The mean static pulmonary compliance in our population was in the normal range (47 ± 16 mL cmH_2_O^−1^), and so an increase in intrapulmonary pressure would have been transmitted to the adjacent compartments. Since patients with right ventricular dysfunction or lung disease were excluded from the study, one can assume that right ventricular function in our study population was normal. Secondly, the increase in intrathoracic pressure caused by continuous PEEP acts on the right ventricle in 2 ways.^[18–21]^ Firstly, this increase is transmitted to abdominal compartment, which increases resistance to venous return due to collapse of the vena cava and the hepatosplanchnic venous circulation.^[18,19]^ However, PEEP increases the right ventricle afterload (resulting from the increase in pulmonary vascular resistance) and right ventricle ejectional impedance.^[20]^ These effects on right ventricular preload and afterload are particularly large when the preload is low.^[18,21]^

All the patients in our study displayed a significant decrease in SV during the RM. SV changes during the RM were higher in responders because the latter were preload dependent. In other words, the hemodynamic effects of the RM on the right ventricle depend on preload status, which was reflected by large changes in SV and arterial blood pressure. This has been observed in earlier studies and may explain the RM's excellent predictive value as a preload dynamic test.^[18–22]^ The ΔrecPP has good predictive value, due to the lesser impact of the increase in intrathoracic and pleural pressures on the arterial vascular compartment, and the dependence between SV and PP. Nevertheless, the broad gray zone may limit the use of ΔrecPP at the bedside.

In 2005, De Backer et al initiated a debate on the relevance of dynamic preload indices when patients were ventilated with low TVs (less than 8 mL kg^−1^).^[8]^ This topic is still subject to debate, with the publication of contradictory finding.^[23,24]^ It is important to take account of the characteristics of the study population (eg, normal vs altered lung compliance), the study setting (operating theater vs intensive care unit) and the monitor used to track CO or SV changes (calibrated vs noncalibrated pulse wave contour analysis, EDM, echocardiography, etc).^[25]^ As mentioned earlier, the magnitude of the pulmonary transmission index submit to the conditions of the effect of intrathoracic pressure on the preloads and afterloads of the right and left ventricles.^[26]^ This suggests that respiratory variations are related to preload dependency—even at low TVs. As the TV decreases, so do respiratory variations in SV and PP; this may reduce the sensitivity and the cut-offs associated with these indices.^[27]^ As mentioned in the literature, the analysis of SV and its changes must take account of the measurement site (the descending thoracic aorta, radial artery or femoral artery) and the measurement device.^[25,27]^ It is likely that measurements at a distal artery (such as the radial artery) have a greater signal/noise ratio due to the damping of the signal along the vascular tree and the low respiratory variations (due to the decrease in TV). This reduces the index's ability to discriminate between fluid responders and non-responders ^[28]^ and may explain in part why ΔrespSV (measured with EDM at the descending thoracic aorta) was predictive of fluid responsiveness but ΔrespPP (measured at the radial artery) was not.

This study had several limitations. Our institution's choice of RM can be debated. In the past, we have found that high intrathoracic pressures have harmful effects on patients without lung disease. Several studies have demonstrated that lower levels of PEEP still provide satisfactory levels of arterial oxygenation, with better hemodynamic tolerance.^[18,22]^ Thus, the level and duration of the positive intrathoracic pressure were chosen to optimize the benefit-risk balance (ie, the balance between alveolar recruitment and potentially harmful hemodynamic effects) in disease-free patients. A recent review by Güldner and colleagues reported great heterogeneity in the RMs and protective ventilation modes used in the operating theatre, and did not provide specific recommendations on RMs.^[29]^ Hence, the use of ΔrecSV at the bedside during hemodynamic optimization can be considered. Even though this parameter was most predictive, it should be used as an adjunct to other parameters of preload dependency (respiratory variables, and SV changes during fluid challenge). Given that repeated RMs can have harmful hemodynamic effects, the use of this approach should be avoided in hemodynamically unstable patients. At the bedside, preload dependency should be assessed as part of a multimodal approach based on a clinical examination, hemodynamic parameters, dynamic preload indices, and the SV change during fluid challenge. This approach may increase the accuracy of diagnosis of preload dependency, especially when the preload indices are in the gray zone. Given that we excluded patients with cardiac arrhythmia, right ventricular failure, spontaneous ventilation, and altered lung compliance, our present results cannot be extrapolated to other patient profiles. Furthermore, the CardioQ EDM system does not measure the aortic diameter, and CO is estimated from the aortic blood flow velocity-time integral. This bias has been discussed in the literature, and a number of studies have demonstrated that ΔrespSV can still be predicted in the absence of an aortic diameter measurement.^[13,15]^ Lastly, one must consider the impact of possible EDM probe displacement during the RM on the measurement of SV. To avoid artifacts related to failure to identify the beginning and end of aortic flow with each ventricular beat, we ensured that laminar flow was present by using a narrow frequency range (ie, a blunt velocity profile) At the bedside, we did not observe any significant changes of the envelope of aortic flow signal during RM. This type of change would have worsened the predictive value of ΔrecSV because of false positives. Since ΔrecSV was of value in predicting fluid responsiveness, we believe that the putative impact of RMs on displacement of the EDM probe was marginal.

## 5 Conclusion

During abdominal surgery with protective ventilation (ie, with PEEP, RMs, and a low TV), ΔrecSV was strongly predictive of fluid responsiveness and had a narrow gray zone. ΔrecPP and ΔrespSV were weakly predictive of fluid responsiveness, and ΔrespPP was not predictive.
